# The anti-bat strategy of ultrasound absorption: the wings of nocturnal moths (Bombycoidea: Saturniidae) absorb more ultrasound than the wings of diurnal moths (Chalcosiinae: Zygaenoidea: Zygaenidae)

**DOI:** 10.1242/bio.021782

**Published:** 2016-12-02

**Authors:** Athanasios Ntelezos, Francesco Guarato, James F. C. Windmill

**Affiliations:** Centre for Ultrasonic Engineering, Department of Electronic and Electrical Engineering, University of Strathclyde, 204 George Street, Glasgow, G1 1XW, UK

**Keywords:** Moth, Saturniidae, Chalcosiinae, Predation-driven sexual dimorphism, Ultrasound absorption, Predator-prey interactions

## Abstract

The selection pressure from echolocating bats has driven the development of a diverse range of anti-bat strategies in insects. For instance, several studies have proposed that the wings of some moths absorb a large portion of the sound energy contained in a bat's ultrasonic cry; as a result, the bat receives a dampened echo, and the moth becomes invisible to the bat. To test the hypothesis that greater exposure to bat predation drives the development of higher ultrasound absorbance, we used a small reverberation chamber to measure the ultrasound absorbance of the wings of nocturnal (Bombycoidea: Saturniidae) and diurnal moths (Chalcosiinae: Zygaenoidea: Zygaenidae). The absorption factor of the nocturnal saturniids peaks significantly higher than the absorption factor of the diurnal chalcosiines. However, the wings of the chalcosiines absorb more ultrasound than the wings of some diurnal butterflies. Following a phylogenetic analysis on the character state of diurnality/ nocturnality in the Zygaenidae, we propose that diurnality in the Chalcosiinae is plesiomorphic (retained); hence, the absorbance of their wings is probably not a vestigial trait from an ancestral, nocturnal form but an adaptation to bat activity that overlaps their own. On a within-species level, females of the saturniids *Argema mittrei* and *Samia cynthia ricini* have significantly higher absorption factors than the males. In the female *S. c. ricini*, the higher absorption factor corresponds to a detection distance by bats that is at best 20-30% shorter than that of the male.

## Abstract (precis)

**Summary:** Moth wings partly absorb the ultrasonic calls of bats to reduce predation. Different moths fly at night or day, and this work compares their absorption of ultrasound.

## INTRODUCTION

The prey-predator interaction between moths and bats has served for decades as an example of a coevolutionary arms race. Moths have developed a series of measures to avoid detection from the echolocation cries of bats (primary defences) and to promote survival once detected (secondary defences). Of all defence mechanisms, ultrasonic hearing is probably the one that has received the most attention (see reviews from Yack et al., 1999; Minet and Surlykke, 2003). Studies have reported at least three response mechanisms of eared moths exposed to echolocation cries or artificial ultrasound. The first is the evasive action of flying moths, which increases their chance of survival 40-50% over sympatric earless species (Acharya and Fenton, 1999; Roeder, 1998). The second is the adaptive silence of the non-aerial male lesser wax moth (*Achroia grisella*) that ceases its courtship song (Greenfield and Baker, 2003; Spangler, 1984). The third is the clicking sounds of tiger moths (Noctuoidea: Erebidae) that function as acoustic aposematism, Batesian or Müllerian mimicry, startle signals, or echolocation jamming (Conner, 2014; Conner and Corcoran, 2012; Miller and Surlykke, 2001).

Hearing clearly offers a survival advantage; however, the auditory system comes with energetic and behavioural costs and when it is no longer beneficial, such as in some moths that escape bat predation, it regresses (Miller and Surlykke, 2001). Several studies have measured directly the high energetic expenditure on the neural tissue of visual sensory systems (Niven and Laughlin, 2008), and although there are no equivalent direct data for auditory systems, the energy cost hypothesis is applicable throughout the nervous system (Niven and Laughlin, 2008). The potential behavioural costs for an eared moth emanate from the evasive response, which can result in the moth landing on a water surface (Guignion and Fullard, 2004) or missing a mating opportunity (Yager, 2012). These could be reasons why some moths under bat predation are deaf, yet rely on non-auditory anti-bat measures, such as their flight behaviour. Earless moths tend to fly less (Morrill and Fullard, 1992; Soutar and Fullard, 2004) or erratically (Lewis et al., 1993), and achieve acoustic crypsis by remaining close above vegetation in order to blend into the echoes of surrounding clutter (Fullard and Napoleone, 2001; Lewis et al., 1993; Rydell, 1998). Other earless species avoid bats more drastically: they emerge during seasons or times of the day when bat activity is low (Lewis et al., 1993; Soutar and Fullard, 2004; Yack, 1988).

Recent research revealed how some moths utilize the morphology of their wings towards two non-auditory defences: acoustic deflection and ultrasound absorption. The first is the acoustic equivalent of the visual deflection that several Lepidoptera species employ to deflect visually guided attacks from their body towards eyespots on their wings (Brakefield and Larsen, 1984; Lyytinen et al., 2003; Olofsson et al., 2010; Stevens, 2005; Vallin et al., 2011). Accordingly, luna moths (*Actias luna*) deflect bat attacks towards their long hindwing tails. Tailed moths have a ∼47% survival advantage over moths that have their hindwing tails ablated, an advantage similar to the one of eared over earless moths (Barber et al., 2015). In addition to diverting attacks, moth wings may absorb a large portion of the energy of an incidental echolocation cry. As a result, the intensity of the reflected echo and the detection distance of the moth decline. Several studies have proposed that the scales on moth wings may increase the absorbance of bat echolocation cries (Moss and Zagaeski, 1994; Roeder, 1963; Zeng et al., 2011). Zeng et al. (2011) designed a novel method for the measurement of absorbance in the ultrasonic spectrum and compared the absorption factors (measured absorption quantity) between the wings of nocturnal moths and diurnal butterflies. The latter absorbed significantly less ultrasound in the frequency range 40-60 kHz, presumably because they are not exposed to bat predation.

Accordingly, we compare the absorption factors between the wings of members of the Saturniidae (Bombycoidea) and of the Chalcosiinae (Zygaenoidea: Zygaenidae). Several studies have reported on the nocturnal activity of the saturniid study species *Argema mittrei* (Guérin-Méneville 1847) (Jolly et al., 1984), *Automeris io* (Fabricius 1775) (Fullard and Napoleone, 2001), and *Samia cynthia ricini* (Jones 1791) (Eguchi et al., 1982). Although there are no studies on the behaviour of the chalcosiines in this study [*Amesia aliris analis* (Jordan 1907), *Campylotes burmanus* (Hampson 1919), and *Erasmia pulchera* (Hope 1840)], the chalcosiines are primarily diurnal (Subchev, 2014; Yen et al., 2005). The purpose of this comparative study is to test the hypothesis that the between-families and between-species absorption factors of wings differ significantly due to different degrees of exposure to bat predation. Moreover, the saturniid study species are sexually dimorphic, and so the within-species absorption factors of males and females are also compared.

## RESULTS

### Measurement of moth wing absorption factors in a small reverberation chamber

**Fig. 1.**
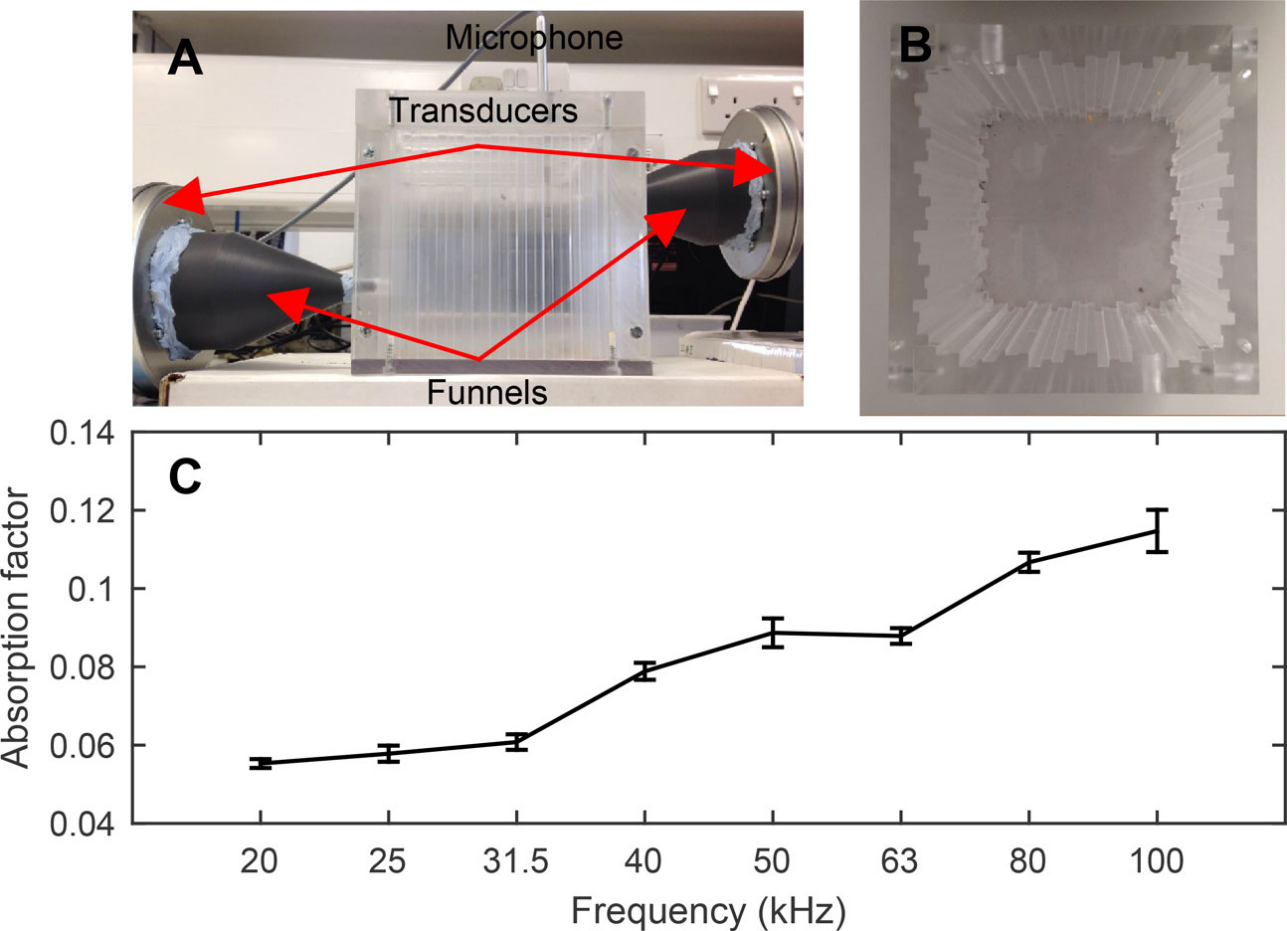
**Small reverberation chamber for the measurement of absorption factors in the ultrasonic spectrum.** (A) Experimental set-up. Funnels direct the ultrasound emitted by the transducers into the chamber through a 7 mm opening. The microphone can move freely up and down inside the chamber, allowing for spatial averaging of the reverberation times. (B) Overview of reverberation chamber showing the engraved diffusers. (C) Absorption factor of empty reverberation chamber (Eqn 1); the error bars indicate 95% confidence intervals (CIs) (*n*=27 microphone positions).

**Fig. 2.**
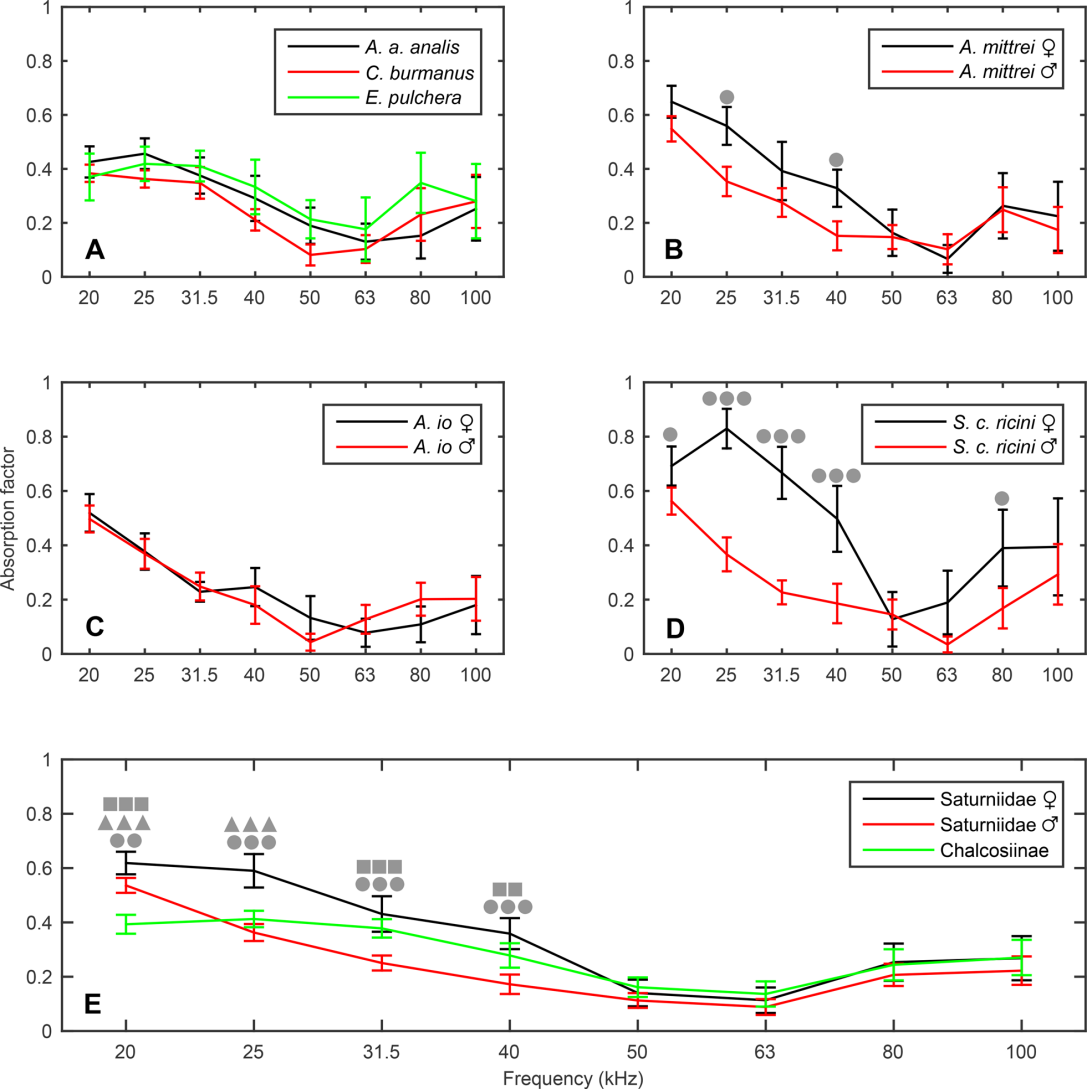
**Absorption factors of moth wings measured with small reverberation chamber (Eqn 3).** (A) Chalcosiines. (B-D) Female and male saturniids. The absorption factors of *A. mittrei* and *S. c. ricini* differ significantly according to sex for the frequencies indicated by circles. (E) Absorption factors grouped by preparation group. The absorption factors differ significantly from each other for frequencies within the range 20–40 kHz. Circles indicate significant difference between male and female saturniids, triangles between female saturniids and chalcosiines, and squares between male saturniids and chalcosiines. All pairwise comparisons were conducted with Tukey-Kramer test for each frequency separately. One, two, or three symbols correspond to 0.01<*P*<0.05, 0.001<*P*<0.01, and *P*<0.001, respectively. The error bars indicate the 95% CIs and *n*=6 sets for each sample.

Dried specimens and a small reverberation chamber (Fig. 1) were used to measure the random incidence absorption factors of the wings of each study species (separately for male and female saturniids) over the frequency range 20–100 kHz (Fig. 2). For each treatment *n*=6 sets were used, with each set comprising the maximum number of non-overlapping moth wings of same-sex specimens of a species that could fit on the reverberation chamber floor. The number of wings of each set depended on species size and availability of dried specimens.

**Fig. 3.**
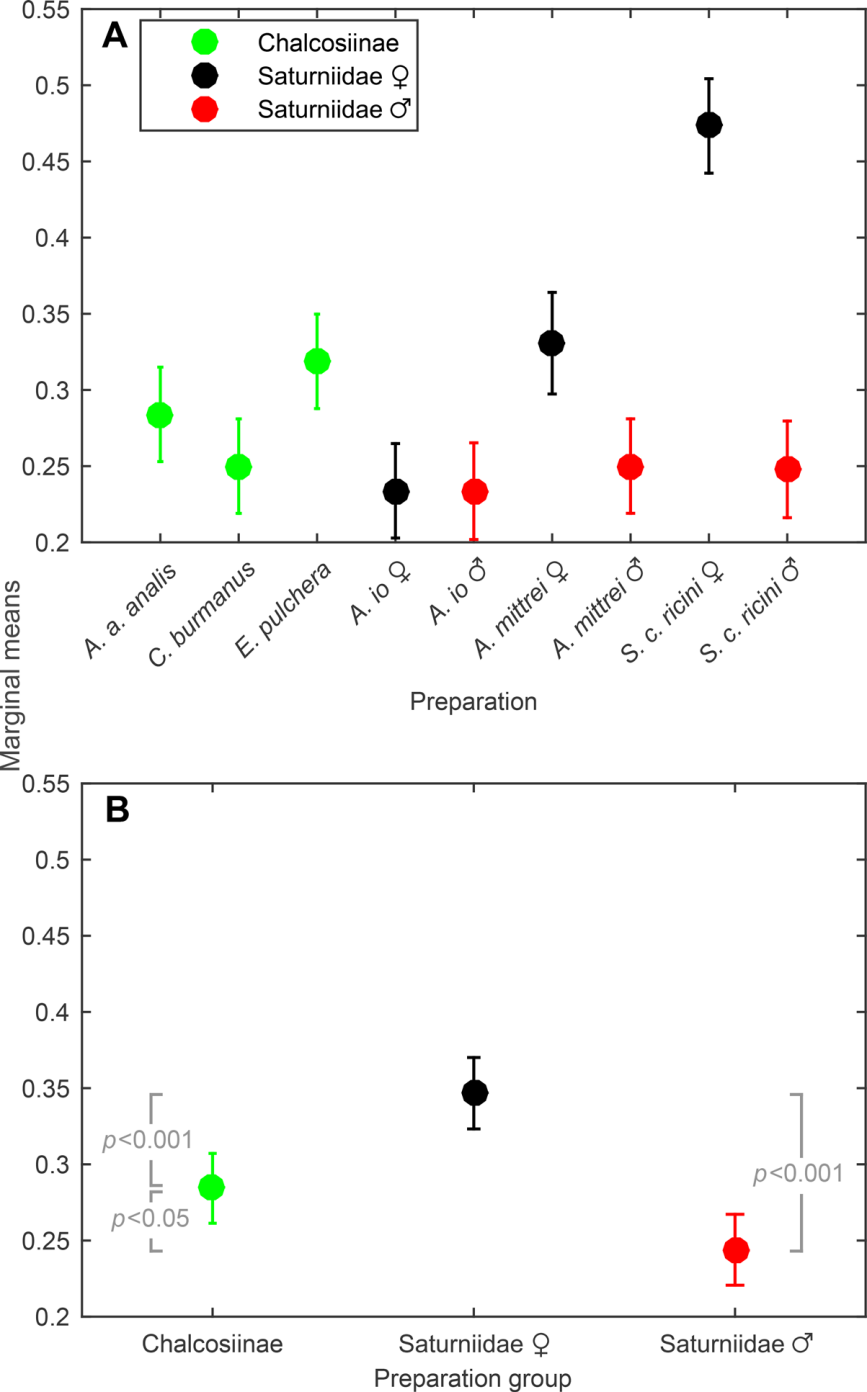
**Estimated marginal means of absorption factors of moth wings across the frequency range 20–100 kHz.** (A) Estimated marginal mean of each preparation. There is a significant effect of preparation on estimated marginal mean (ANOVA, *F*_8,45_=23.78, *P*<0.001). (B) Estimated marginal means grouped by preparation group. The estimated marginal means differ significantly according to preparation group (ANOVA, *F*_2,51_=19.08, *P*<0.001) and also differ significantly from each other (Tukey-Kramer test). The error bars show the 95% CIs [*n*=48 in A (6 sets×8 frequencies) and *n*=144 in B (3 preparations×6 sets×8 frequencies)].

The absorption factors of all preparations follow similar patterns, with a peak occurring within the frequency range 20–25 kHz (Fig. 2A-D). However, there is a significant effect of preparation on absorption factor, meaning that the estimated marginal means across the frequency range 20–100 kHz differ significantly according to preparation (ANOVA, *F*_8,45_=23.78, *P*<0.001; Fig. 3A). Apart from preparation, frequency also has a significant effect on absorption factor, signifying that the absorption factor is frequency dependant (repeated measured ANOVA, *F*_7,315_=129.13, *P*<0.001; *P*-value computed with Greenhouse-Geisser correction). Furthermore, there is a significant preparation-frequency interaction indicating that different preparations have different absorption factors at different frequencies (repeated measures ANOVA, *F*_56,315_=3.87, *P*<0.001; *P*-value computed with Greenhouse-Geisser correction). Pairwise comparisons showed that the absorption factors of the chalcosiines do not differ significantly from each other (Tukey-Kramer test, *A. a. analis*–*C. burmanus*, *P*=0.842; *A. a. analis*–*E. pulchera*, *P=*0.823; *C. burmanus*–*E. pulchera*, *P=*0.051). However, when it comes to within-species comparisons in saturniids, it appears that the absorption mechanisms of *A. mittrei* and *S. c. ricini* are sexually dimorphic; although the absorption factors between male and female *A. io* do not differ significantly (Tukey-Kramer test, *P*<0.05, *P*<0.001, and *P*=0.99 respectively). Specifically, the between-sexes absorption factors of *A. mittrei* and *S. c. ricini* differ significantly for frequencies within the range 20–40 kHz (Tukey-Kramer test for each frequency separately, see Fig. 2B and D for significant *P-*values), where females have higher absorption factors.

To compare by preparation group, the absorption factors of each group were calculated (Fig. 2E). The estimated marginal means of the absorption factors across the frequency range 20–100 kHz differ significantly according to preparation group (ANOVA, *F*_2,51_=19.08, *P*<0.001; Fig. 3B), and pairwise comparisons show that they significantly differ from each other (Tukey-Kramer test, Chalcosiinae–female Saturniidae, *P*<0.001; Chalcosiinae–male Saturniidae, *P*<0.05; female Saturniidae–male Saturniidae, *P*<0.001). The female saturniids have the highest estimated marginal mean, followed by the chalcosiines and the male saturniids. Apart from the significant group effect on absorption factor, there is a significant effect of frequency on absorption factor as well as a significant group-frequency interaction (repeated measures ANOVA, *F*_7,357_=123.13, *P*<0.001 and *F*_14,357_=7.46, *P<*0.001 respectively; *P*-values computed with Greenhouse-Geisser correction). Pairwise comparisons grouped by frequency show that the differences of each pair of preparation groups lie within frequency range 20–40 kHz (Tukey-Kramer test for each frequency separately, see Fig. 2E for significant *P*-values). The female saturniids generally have higher absorption factors than the other two preparation groups for this frequency range, and while the male saturniids have a significantly higher peak than the chalcosiines, their absorption factors decline more steeply, hence the abovementioned higher marginal mean of the chalcosiines.

### Survival advantage of female *S. c. ricini* over males in terms of smaller detection distance by bats

**Fig. 4.**
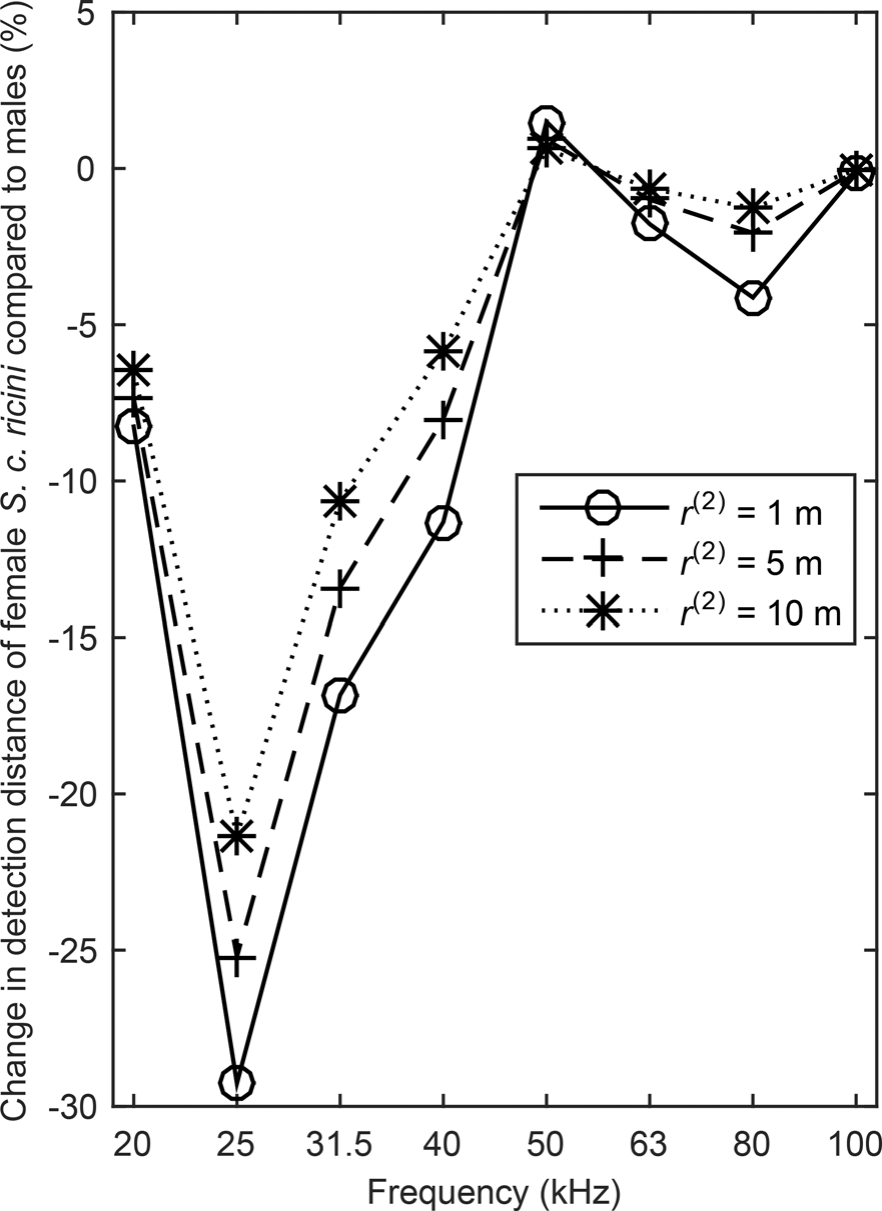
**Percentage difference in the detection distance of female *S. c. ricini* compared to three hypothetical detection distances of male *S. c. ricini* [r^(2)^].** The difference between the detection distances of male and female *S. c. ricini* is maximized at 25 kHz, where the difference between the absorption factors of their wings is also maximized (Fig. 2D). The detection distances of female *S. c. ricini* were calculated with Eqn 13 for atmospheric attenuation at temperature 20°C and 70% relative humidity (Surlykke, 1988).

The detection distance of a target by a bat depends on the transmission loss due to spherical spreading and atmospheric attenuation, and on the target strength (Møhl, 1988). The detection distances of male and female *S. c. ricini* were compared because they have wings of similar size and shape; consequently the comparison is feasible because their target strengths, and hence their detection distances, differ due to the different absorption factors of their wings alone*.* Detection distances of moths typically range between 1–10 m (Surlykke et al., 1999). Eqn 13 was used with three hypothetical detection distances within this range for male *S. c. ricini* in order to derive the respective detection distances of female *S. c. ricini* (Fig. 4). At 25 kHz, where the difference between the absorption factors of male and female *S. c. ricini* is maximized (Fig. 2D), the detection distance of female *S. c. ricini* is 20–30% smaller.

## DISCUSSION

Studies have reported bat echolocation calls with dominant frequencies ranging from 11 kHz (*Euderma maculatum*; Fullard and Dawson, 1997) to 212 kHz (*Cloeotis percivali*; Fenton and Bell, 1981), though most insectivorous bats echolocate with dominant frequency 20–60 kHz (Fenton et al., 1998). The ultrasound absorbance of the wings of this study's moth species peaks at the lower end of this range (20–25 kHz; Fig. 2). Despite the similar patterns, there are significant differences not only between the nocturnal saturniids and the diurnal chalcosiines (Fig. 2E), but also between male and female *A. mittrei* (Fig. 2B) and *S. c. ricini* (Fig. 2D).

Zeng et al. (2011) proposed that the scales on the wings are responsible for the ultrasound absorption. The scales have interstitial spaces between them that create a network of interconnected pores similar to that found in porous sound absorbers (Zeng et al., 2011); when a sound wave propagates through this network, thermal and viscous effects cause the dissipation of its acoustic energy (Cox and D'Antonio, 2009a). In addition, the ultrastructure of the scales resembles a perforated panel backed by air (Zeng et al., 2011), which could act as a microperforated panel absorber (Cox and D'Antonio, 2009b).

The absorption of ultrasound is not the sole defensive function of the moth wing scales. For instance, a moth can release itself from a spider web by shedding some of its wing scales (Eisner et al., 1964). Furthermore, the microstructure of the scales is responsible for some of the colours found in moth wings (Brink and Lee, 1996; Ghiradella, 1991). In saturniids, the wing colours and patterns can play a defensive role by achieving crypsis, aposematism, or mimicry (Blest, 1957a,b), and in *Callosamia securifera*, *C. promethea*, and *Eupackaria calleta*, only the males are Batesian mimics of the unpalatable swallowtail *Battus philenor* and/or *B. polydamas* (Jeffords et al., 1979; Sternburg et al., 1977; Waldbauer and Sternburg, 1975). Such sexual dimorphism may have been driven by a more intense selection pressure on males by visual predators (Allen et al., 2011), because the males fly during the day in search of the sedentary, pheromone-releasing females (Morton, 2009).

Accordingly, we hypothesized that male saturniids will exhibit adaptive increase to bat predation compared to females, however, the female *A. mittrei* and *S. c. ricini* exhibit significantly higher absorption factors over certain frequencies (Fig. 2B and D). In the female *S. c. ricini*, the higher absorption factor translates to a detection distance by bats that is 20–30% shorter than the detection distance of the male, assuming a bat echolocates with a dominant frequency of 25 kHz (Fig. 4). Since the absorption mechanism serves no other function but to render the moth inconspicuous to echolocating bats, our results indicate that the female *A. mittrei* and *S. c. ricini* may be under more intense selection pressure by bats than males. Yet further studies on the behaviour of these two species are required to corroborate this hypothesis. These would not be the first examples of sexual dimorphism due to differential bat predation on the two sexes. The males of some species of moths and mantids are under heavier selection pressure from echolocating bats compared to the females, and as a result they have developed more sensitive ultrasonic hearing (moths: Cardone and Fullard, 1988; Rydell et al., 1997; mantids: Yager, 1990). However, it is possible that the absorption factors do not vary between the sexes as predicted because there are trade-offs among the functions of the wing scales. In that case, intense selection for e.g. the colour-producing properties of the wing scales (Brink and Lee, 1996; Ghiradella, 1991) of males would undermine their ultrasound absorption properties.

The second significant finding of our study regards the differences between the absorption factors of the nocturnal saturniids and of the diurnal chalcosiines. Comparative studies on moth ears that function as bat detectors have reported on the degenerate state of audition in diurnal moths compared to nocturnal ones. Such findings suggest that diurnal activity could be an apomorphic (derived) trait that has allowed some moths to escape bat predation (Fullard et al., 1997; Muma and Fullard, 2004). Accordingly, we hypothesised that if the ultrasound absorption mechanism is the result of bat predation pressure, then the diurnal chalcosiine study species should have significantly lower absorption factors compared to the nocturnal saturniids. Indeed, the absorption factor of the chalcosiines peaks at approximately 0.4, whereas the absorption factors of the male and female saturniids peak around 0.6 (Fig. 2E). However, the peak of 0.4 may be significant compared to the peaks of 0.1–0.2 reported for butterfly wings and nocturnal moth wings without scales (Zeng et al., 2011). An explanation is that diurnality is an apomorphic trait in chalcosiines; hence, their absorption mechanism is a case of evolution in reverse (Porter and Crandall, 2003). This means that the absorption mechanism is a vestigial trait that the chalcosiines have retained from their ancestral, nocturnal form. The peak absorption factor that is higher than in butterflies but lower than in nocturnal moths may signify that the absorption mechanism has degenerated, but not enough evolutionary time has passed for it to disappear completely. It is possible however that the difference in peak absorbance between the diurnal chalcosiines and the nocturnal saturniids is not due to degeneration of the absorption mechanism, but due to an adaptive increase of the latter to the ongoing selection pressure from bats. Consequently, the chalcosiines could be a window to the absorption mechanism in its primitive form. In cases such as this one, where a vestigial trait persists, there is probably no intense selection pressure against the trait, which may eventually disappear by genetic drift. A relevant example is the fully functioning or degenerate hearing of some moths that are completely isolated from bats, spatially or temporally (review: Miller and Surlykke, 2001).

The hypothesis that diurnality in chalcosiines is apomorphic was tested with phylogenetic analysis, by mapping the character state of nocturnality/ diurnality on the phylogenetic tree of the Zygaenidae (Niehuis et al., 2006). Of the four zygaenid subfamilies, the Zygaeninae, the Procridinae, and the Chalcosiinae are predominately diurnal, at least when it comes to mating, but there are not enough data to make a conclusion regarding the Callizygaeninae (Subchev, 2014). Since three out of the four zygaenid subfamilies are diurnal, it is highly probable that diurnality is a pleisiomorphic (ancestral), not apomorphic, trait in the Chalcosiinae. Therefore, the fact that the peak absorbance of chalcosiine wings is higher than that of butterfly wings (Zeng et al., 2011) requires an alternative explanation.

Fullard et al. (2000) suggested that the terms ‘diurnal’ and ‘nocturnal’ can be misleading, especially when describing diel flight activity at a higher taxonomic level (e.g. subfamily). Diurnality and nocturnality are not mutually exclusive, and most so-called diurnal moths exhibit mixed diurnal/nocturnal flight activities (Fullard and Dawson, 1999; Fullard and Napoleone, 2001; Fullard et al., 2000), though some are exclusively diurnal (Fullard et al., 2000; Muma and Fullard, 2004). The fact that the Chalcosiinae are well adapted to diurnal activity – their mimetic and aposematic wing patterns deter predators that hunt using visual cues (Endō and Kishida, 1999; Owada and Tinh, 2002) – does not preclude them from potential nocturnal activity, and thus exposure to bat predation. Ultimately, considering the phylogenetic analysis, it is more likely for the absorption mechanism of the chalcosiines to be an adaptive measure to counter occasional bat encounters than to be an apomorphic, vestigial trait. On the other hand, butterflies tend to be exclusively diurnal (Fullard and Napoleone, 2001; Fullard et al., 2000), a fact that is reflected by the very low absorption factors of their wings (Zeng et al., 2011).

## MATERIALS AND METHODS

### Dried specimens

Male and female saturniid specimens (*A. io* from USA, *A. mittrei* from Madagascar, and *S. c. ricini* from China) and male chalcosiine specimens (*A. a. analis* and *C. burmanus* from Thailand, and *E. pulchera* from Burma) were obtained from the Lepidoptera Breeders Association (Bourne, UK).

### Measurement of moth wings absorption factor with the reverberation chamber method

The time period for which it takes the sound pressure level to drop 60 dB after a sound stops is termed reverberation time and depends on the total absorption inside a reverberation chamber. Consequently, by comparing the reverberation time before and after the introduction of an absorbent material (e.g. moth wings) inside the chamber, the random incidence absorption coefficient of the introduced material can be derived. The standard for the measurement of the random incidence absorption coefficient in a reverberation chamber essentially concerns frequencies below 20 kHz (ISO, 2003). For this frequency spectrum, the measurement procedure requires large chambers and 10-12 m^2^ of absorbent material (Cox and D'Antonio, 2009c); however, such a quantity of moth wings is practically infeasible. Besides, the shorter wavelengths of the ultrasonic spectrum render feasible the measurement of the absorption coefficient with a smaller chamber and smaller quantities of absorbent material. Still, since this study did not follow the standard faithfully, the measured absorption quantity has been called absorption factor. The same term was employed by the only other documented study of sound absorption in the ultrasonic spectrum (Zeng et al., 2011).

A small reverberation chamber for ultrasonic applications (Fig. 1) was fabricated for the measurement of the random incidence absorption factors of the moth wings. The objective of the reverberation chamber is to create a spatially uniform acoustic field where the sound pressure level is ideally the same for every point within the chamber. In order to achieve the diffuse field, an acoustic wave should be equally probable to propagate towards any direction. For this reason, Cox and D'Antonio modified primitive root diffusers (Cox and D'Antonio, 2000), which promote diffusion of the incident wave towards all directions and reduce the number of standing waves, and these were engraved on the walls and ceiling of the chamber (Fig. 1A,B). In addition, a reverberation chamber must have hard, non-absorbing surfaces that reflect most of the acoustic energy back into the chamber. Our reverberation chamber was fabricated with Polymethyl 2-methacrylate (Perspex) that secured low ultrasound absorbance (Fig. 1C).

Even with these measures, the reverberation time is spatially dependent within the reverberation chamber. To reduce the effect of non-diffuseness, two ultrasonic transducers equipped with funnels were utilised to direct the ultrasound into the chamber through 7 mm openings, scattering multi-directionally the acoustic wave that entered the chamber. In addition, the recording device, an ultrasonic microphone, could move freely up and down inside the chamber, allowing for spatial averaging of the measured reverberation times (Fig. 1A).

The absorption factor of the empty chamber was determined by (Cox and D'Antonio, 2009c):
(1)
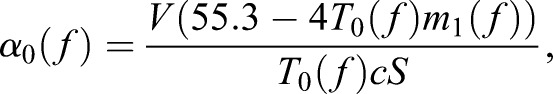
where *α*_0_ is the average absorption factor of the empty chamber, *V* (m^3^) is the volume of the chamber, *f* (Hz) is the sound frequency, *T*_0_ (s) is the reverberation time before the introduction of moth wings inside the chamber, *m*_1_ (m^−1^) is the air volume absorption coefficient, *c* (m s^−1^) is the speed of sound in the air, and *S* (m^2^) is the surface area of the chamber.

The absorption factors of the empty chamber and of the moth wings were measured over nine 1/3 octave bands that have centre frequencies from 20 to 100 kHz. The parameter *m*_1_ was calculated by:
(2)
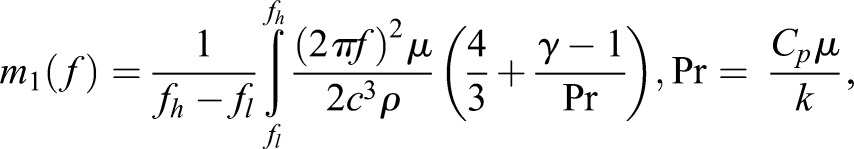
where *f_l_* and *f_h_* (Hz) are the low and high frequency limits of the 1/3 octave band, *μ* (Pa s) is the dynamic viscosity of air, *ρ* (kg m^−3^) is the density of air, *γ* is the ratio of specific heats of air, Pr is the Prandlt number of air, *C_p_* (J kg^−1^ K^−1^) is the heat capacity of air at constant pressure, and *k* (W m^−1^ K^−1^) is the thermal conductivity of air (Table S1).

The absorption factors of the moth wings were determined by (Cox and D'Antonio, 2009c):
(3)
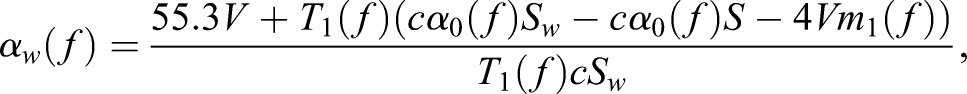
where *α_w_* is the absorption factor of the moths wings introduced inside the chamber, *S_w_* (m^2^) is their surface area, and *T*_1_ (s) is the reverberation time after the introduction of the moth wings.

For the measurement of a reverberation time with respect to frequency *f*, a function generator (Agilent 33220A) generated a sinusoidal signal of frequency *f* that was amplified with an amplifier (Ultrasound Advice S55) before transmission inside the chamber by two ultrasonic transducers (Ultrasound Advice S56); the transmission stopped when the response inside the chamber reached the steady state. The signal was recorded with a microphone (Bruel & Kjaer 4138) using a sampling frequency of 625 kHz and was amplified with a pre-amplifier (Nexus 2690). The reverberation times were derived from the steady state decay curves. To determine *α*_0_(*f*) (Fig. 1C), *T*_0_(*f*) was measured from *n*=27 microphone positions for each 1/3 octave band with centre frequency *f*. To determine *α_w_*(*f*) (Fig. 2), *n*=6 sets per treatment were used and *T*_1_(*f*) of each set was averaged from four microphone positions. The term set refers to each set-up of non-overlapping moth wings of same-sex specimens of a species that cover the maximum possible surface area of the reverberation chamber floor. The number of wings of each set depended on species size and availability of dried specimens.

### Estimation of moth wings surface area

*S*_w_ was estimated with an image processing method (Fig. S1). The wings of each set were placed on a white background of known surface area and an image was obtained. The image was smoothened with edge-preserving bilateral filter (Tomasi and Manduchi, 1998) and was subsequently converted to binary via k-means++ clustering (Arthur and Vassilvitskii, 2007). In the binary image, one class of pixels represents the wings and the other represents the background. *S_w_* was estimated with
(4)
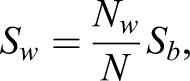
where *N_w_* is the number of pixels representing moth wings, *N* the total number of pixels, and *S_b_* the surface area of the white background.

### Derivation of reverberation time from steady state decay curve

First, the recorded signal of main frequency *f* was filtered with a bandpass digital filter designed with a Kaiser window. The filter passed frequencies between *f_l_* and *f_h_* in order to retain only frequencies within the 1/3 octave band, frequencies outside this range were attenuated 80 dB (Fig. S2A).

The decay curve of the filtered signal has many fluctuations that render difficult the identification of the point where the response drops 60 dB after the offset of sound; therefore, the curve has to be smoothed. The first step of the smoothing process is to obtain the filtered signal's envelope by using the Hilbert transform:
(5)
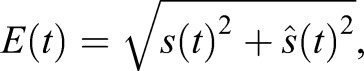
where *t* (s) is the time, *s* (V) is the signal, *s*^ (V) is its Hilbert transform, and *E* is its envelope. The envelope was further smoothed with a 625-samples long moving average filter that corresponds to a time interval of 1 ms (Fig. S2B).

The steady-state reverberation time was derived from the smoothed curve by Schroeder's integration (Schroeder, 1965):
(6)
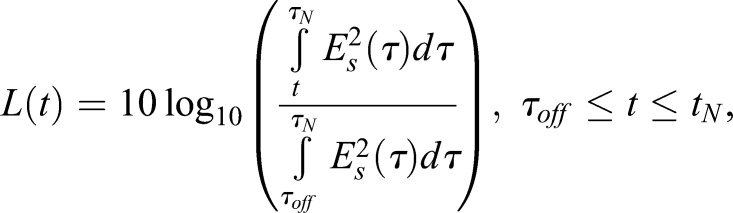
where *L* [dB] is Schroeder's curve, *E_s_* (V) is the smoothed envelope, *τ_off_* (s) is the time point where the sound stops, and *τ_N_* (s) is the time point where the signal merges with the noise level. The parameter *τ_N_* was estimated with the method proposed by Lundeby et al. (1995).

*L* is a maximally flat curve, meaning that its gradient is constantly negative. As a result, the curve crosses the −60 dB point once. This property makes Schroeder's integration method useful for the estimation of the reverberation time. However, in most cases L(*τ_N_*)> –60[dB]. This means that the signal merges with the noise level before reaching the −60 dB point. Therefore, the reverberation time has to be estimated using linear regression. *L* is approximated with the linear model, *L΄*(*t*)=*A·t*+*B*, and the reverberation time can then be estimated with
(7)
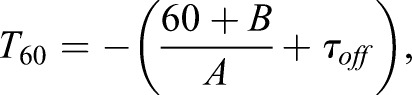
where *T*_60_ is the reverberation time (Fig. S2C).

### Statistical analysis

To compare the absorption factors among the nine preparations (3 chalcosiines and 3×2 male and female saturniids; Fig. 2A-D and Fig. 3A), a repeated measures model, specifically a subject-by-treatment model (Doncaster and Davey, 2007), was fitted. In the model, the absorption factors are the responses, preparation is the between-subjects factor, which is used as the predictor variable, and frequency is the within-subject factor. ANOVA was used to test if the absorption factors differ significantly according to preparation (Fig. 3A), and repeated measures ANOVA to test if there is a significant effect of frequency on absorption factor as well as significant preparation-frequency interaction. The *P*-values of the repeated measures ANOVA were computed using a Greenhouse-Geisser correction (*ε*=0.79) because Mauchly's test for sphericity indicated that that the sphericity, hence the compound symmetry assumption, does not hold (χ^2^=144.80, *P*<0.001). To do pairwise comparisons between preparations, post hoc Tukey-Kramer tests were used (Fig. 2A-D and Fig. 3A).

Accordingly, a repeated measures model was used for the comparisons among the three preparation groups (chalcosiines, male and female saturniids; Fig. 2E and Fig. 3B), albeit with the preparation group as the between-subjects factor. The same tests were carried out as with the above model. Again, the sphericity did not hold (χ^2^=142.61, *P*<0.001), and a Greenhouse-Geisser correction (*ε*=0.81) was used for the calculation of the repeated measures ANOVA *P*-values. All statistical analysis was conducted in MATLAB (Mathworks, UK), and a *P*-value of <0.05 was considered statistically significant.

### Survival advantage of female *S. c. ricini* over males in terms of smaller detection distance by bats

The relationship between the detection distances of male and female *S. c. ricini* was derived using the sonar equation (Møhl, 1988):
(8)
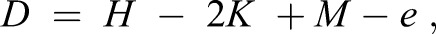
where *D* is the auditory detection threshold of the bat, *H* is the source level, *K* is the one-way transmission loss, *M* is the target strength, and *e* is the noise. The equation is in dB form and all quantities are measured in dB.

*K* is given by:
(9)
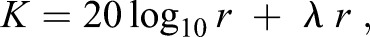
where *r* (m) is the detection distance of the target (e.g. moth), and *λ* (dB m^−1^) is the atmospheric attenuation factor. The first term of the equation accounts for loss due to spherical spreading and the second one for loss due to atmospheric attenuation.

*M* is given by:
(10)
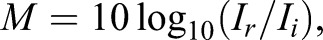
where *I*_*r*_ and *I*_*i*_ (W m^−2^) are the returned and incident sound intensities respectively.

The ratio of incident to returned sound intensity and the absorption factor of the moth wings are related as follows (Cox and D'Antonio, 2009c):
(11)
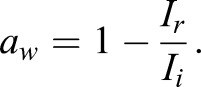
Since male and female *S. c. ricini* have wings of similar size and shape, their ratios of incident to returned sound intensity depend on the absorption factors of their wings alone. Specifically, they are related as follows:
(12)
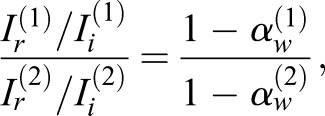
where the superscripts (1) and (2) denote quantities for female and male *S. c. ricini*, respectively.

Expressing Eqn 8 for female *S. c. ricini* and subtracting the respective equation for male *S. c. ricini*, then solving the resulting equation with respect to *r*^(1)^, gives:
(13)
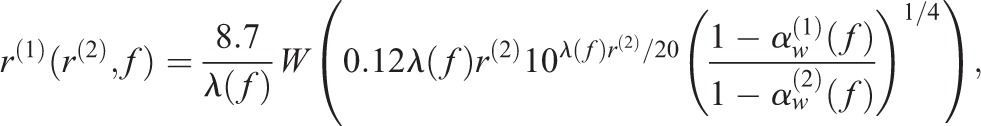
where *W*() is the Lambert W function (Corless et al., 1993, 1996), which in this study returns exactly one real solution. Eqn 13 was used to compare the detection distances of female and male *S. c. ricini* over bat sonar frequency range 20–100 kHz (Fig. 4).
